# The increase of firearm-related violence in Sweden

**DOI:** 10.1080/20961790.2017.1314896

**Published:** 2017-05-03

**Authors:** Ardavan Khoshnood

**Affiliations:** a Department of Criminology, Malmö University, Malmö, Sweden; b Department of Clinical Sciences Lund, Skane University Hospital, Emergency and Internal Medicine, Lund University, Lund, Sweden

**Keywords:** Forensic science, firearms, homicide, gun violence, Skane, Malmo, Sweden

## Abstract

Firearm-related violence is common in our contemporary world and causes serious harm to humans as well as to the society. One of the countries in which firearm-related violence is increasing is Sweden and its most southern region, Skane, in which Malmo, Sweden's third largest city, is highly affected. If not contained and limited, Sweden risks becoming more and more violent.

## Background

Firearm-related violence is a serious threat to mankind and a well-known public health issue [1]. More than 1 000 people are killed by firearms each day [2], as more than 800 million firearms circulate around the world [3]. In discussing criminality and firearm-related violence, a report by the Geneva Declaration [4] states that close to 60% of all global homicides are committed by a firearm, and that only 19% of all homicides in the western Europe is committed by a firearm, which makes it the lowest in the international community. One of the countries in the western Europe usually deemed safe and quite preserved from firearm-related violence and deadly violence has been Sweden. This is, however, no longer true, and even Sweden is today to a high degree affected by the increasing use of firearm-related violence and criminality.

## The case of Sweden

Although highly inconsistent and changeable through the years, deadly violence in Sweden highly increased in 2015 (*n* = 112) [5] in comparison with previous years in which the statistics of deadly violence varied between 81 cases in 2011 [6] and 87 cases in 2014 [7]. 2012 witnessed one of the lowest rates of deadly violence in the country with 68 cases [8]. The statistics for 2016 have not yet been reported by the Swedish National Council for Crime Prevention.

Perhaps not so surprising, the three largest cities in Sweden, Stockholm, Gothenburg and Malmo, are the ones most affected by crime and firearm-related violence. In 2015, the Swedish National Council for Crime Prevention concluded in a report that firearm-related violence is increasing in Sweden [9], not least in south Sweden where Malmo, the country's third largest city lies. Although knife/sharp weapon remains the most common used weapon in an act of violence with respect to *modus operandi*, firearms have been shown to kill more [10], especially in south Sweden [11].

In a study which we recently published from a large city in Sweden on offenders being found guilty for committing homicide and attempted homicide, we could show that firearm was used in more than 35% of the 19 cases which was studied. Even though the use of firearms was less than the use of knife/sharp weapons, firearms to a greater degree contributed to the death of a victim [10]. Comparing this study to other studies from European countries as well as Scandinavian countries [12,13], inclusive of previous Swedish studies [12,14], the use of firearm-related violence is higher and thus troublesome.

The reason for the increase of firearm-related violence in Sweden is probably binary: (1) there is a clear increase of local gangs and gang-related criminality in Sweden where the use of firearm is the most common *modus operandi* [10,15–18], and (2) it is easy for gangs to acquire a firearm because of its widespread in the society [11]. According to the Swedish Police and the Swedish Customs, illegal weapons including firearms and grenades are smuggled into Sweden primary from countries in the West Balkans [19].

## Gun laws in Sweden

Sweden is a country with restrictive gun laws, which is reflected in the low amount of accidental firearm fatalities in the country [20]. In order to own a firearm, a licence must be obtained from the police. After applying for the ownership of a firearm, an extensive investigation will be made by the police about the applicant who must be at least 18 years old and not previously convicted for a crime. The applicant must also clearly state for what purpose he needs to own a firearm and provide necessary documentations for it; the most common reasons are either membership in a shooting club or because of hunting purposes after receiving hunting license first.

The law which foremost regulates the ownership of a firearm is the Swedish Weapon Law from 1996 as legislated by the Swedish Parliament [21]. Further regulations are stated in the weapon decree also from 1996 by the Department of Justice [22], as well as the National Police Board's regulations on the Weapon Law [23].

## Malmo – Sweden's third largest city

Most affected by firearm-related violence in Sweden is the southern region of Sweden, Skane and its largest city Malmo [11]. For the last years, not least 2011/2012 and 2016, Malmo has had serious problems with firearm-related violence and homicides. In 2011/2012, the police were finally able to stop the wave of murders which had taken Malmo like a hostage, after implementing operation Alfred in which the Malmo Police Department was highly supported by the Swedish National Police with extra police officers and detectives [24]. 2016 marked yet another dark year for Malmo, as the city witnessed more than 10 cases of homicides and 20 cases of attempted homicide. If 2016 seemed like a bad dream for the city of Malmo with a population of only 300000, 2017 would in its first 13 days show to be a nightmare. Only three days into the New Year an 18-year-old girl was wounded after being shot [25]. Later the same day a young man was brutally shot down and murdered [26]. Three days later, January 6, a man was wounded after being shot in an apartment [27]. The tragedy culminated on January 12, when a 16-year-old child was viciously shot and killed with several bullets in his head and face as he was on his way home [28]. The police have deemed the shootings to be gang-related or at least connected to gang members.

## The future of Sweden

There is no doubt that firearm-related violence is increasing in Sweden, causing serious consequences for the country, not least in discussing the faith and trust of the people for the police and the judicial system. The phenomenon of gang-related violence in which firearms have a central role [10,29] is new for Sweden. This may very well explain the large problems the Swedish judicial system has in combating firearm-related violence, considering that it is increasing [15–18] and that the great majority of gang-related homicides remains unsolved, even though the police state that they in many cases know who the killer is but cannot prove it [30]. However, with what we are seeing today in Sweden and not least Malmo, serious considerations must be made in reforming the countries judicial system. There is a widespread political unity between the countries political parties in that measures must be taken in order to combat the increasing gang-related violence in the country, but what measures to take and prioritize continues to be a point of discussion.

The Swedish Police are in acute need of more personnel as they today find themselves in a crisis as more police officers than ever before leave their jobs because of low salaries and troubled leadership [31]. Furthermore, in order to combat the increasing gang criminality, a more effective criminal intelligence organization is needed to map and identify elements of interest. As gang criminality is a new phenomenon in Sweden, more training and education is needed for police officers, detectives and investigators. At the same time, we are also in need of more serious and harsher punishments for firearm-related violence. Today, if a person concealing a firearm is arrested by the police, he or she will probably after identification, be set free, without being detained and await trial. The Swedish government should consider a change in the law so that individuals arrested with a firearm can automatically be detained until trial, and not set free. Without acute reforms, Sweden risks becoming more violent as the cycle of violence will never be closed, and the criminal climate will continue to harden.
